# Mechanical properties of CAD/CAM-fabricated in comparison to conventionally fabricated functional regulator 3 appliances

**DOI:** 10.1038/s41598-021-94237-x

**Published:** 2021-07-19

**Authors:** Christoph Roser, Lutz D. Hodecker, Chris Koebel, Christopher J. Lux, Dorothee Ruckes, Stefan Rues, Andreas Zenthöfer

**Affiliations:** 1 Department of Orthodontics and Dentofacial Orthopaedics, Heidelberg University Hospital, Im Neuenheimer Feld 400, Heidelberg, Germany; 2 Department of Prosthodontics, Heidelberg University Hospital, University of Heidelberg, Im Neuenheimer Feld 400, Heidelberg, Germany

**Keywords:** Mechanical engineering, Craniofacial orthodontics, Three-dimensional imaging

## Abstract

Manufacturing of Fränkel's functional regulator 3 (FR3) is complicated and requires extensive knowledge from the dental technician. To determine whether FR3s produced by CAD/CAM techniques (CAD-FR3) meet similar mechanical properties like conventional FR3s (Con-FR3), for each of 10 patient cases, three CAD-FR3 designs (palatal connector cross-section 3 × 3 mm, 4 × 1 mm or 5 × 2 mm) and one Con-FR3 were subjected to cyclic loading and subsequent fracture testing in a universal testing device. Transversal load capacity (F_max(FR3)_) and stiffness were compared among the different CAD-FR3 designs and Con-FR3s using Friedman and Wilcoxon tests with a significance level of α = 0.05. All CAD-FR3 designs had significantly higher mean F_max(FR3)_ (*p* ≤ 0.007) and stiffness (*p* ≤ 0.005) than the Con-FR3s. The CAD-FR3_3×3_ had the highest mean F_max(FR3)_ (98.2 ± 26.2 N) and stiffness (37.1 ± 15.5 N/mm), closely followed by the CAD-FR3_5×2_ (F_max(FR3)_: 90.3 ± 24.7 N; stiffness: 30.0 ± 12.3 N/mm). Among the CAD appliances, CAD-FR3_4×1_ had the lowest values (*p* ≤ 0.007 for all pairwise tests) with F_max(FR3)_ of 45.8 ± 17.9 N and stiffness of 12.5 ± 7.3 N/mm. CAD-FR3s have superior mechanical properties in comparison to Con-FR3s if certain design parameters are followed. Further clinical investigations have to examine if they can serve as an alternative in practice.

## Introduction

Many processes in orthodontics are already carried out digitally, and the proportion is increasing. Examples in diagnostics include digital cephalometric analysis and digital model analysis^1, 2^. In addition, computer-aided design and computer-aided manufacturing (CAD/CAM) technology, which has been applied in prosthetic dentistry for many years^3–5^, is increasingly being used in orthodontics for the production of treatment appliances. The most well established orthodontic CAD/CAM appliances are aligners^6, 7^. Strictly speaking, however, standard aligners are only partially CAD/CAM produced, because the aligners themselves must be produced manually on printed models. Individual studies show direct printing of aligners^8^, but, to date, clinical implementation does not yet seem sufficiently possible with existing printing materials^9^. More examples include customised brackets^10, 11^, bonding trays^12–14^, CAD/CAM anchoring appliances^15^ and fixed CAD/CAM retainers^16^. Other experimental approaches, such as customised CAD/CAM archwires, have been described in individual studies^17^; however, their use is not yet commonplace.

In addition to aligners, other removable appliances can be produced by means of CAD/CAM processes. CAD/CAM production of bite splints, for example, is now very common. Examples include splints for stabilisation after orthognathic surgery^18^, and mandibular-advancement and Michigan splints for the treatment of obstructive sleep apnoea syndrome and temporomandibular disorders, respectively^19, 20^. In contrast, only a few studies describe CAD/CAM production of functional orthodontic appliances, which are used in growth-modification treatment^21, 22^. No study has described CAD/CAM techniques in relation to Rolf Fränkel’s functional regulator 3 (FR3). However, the FR3 could in fact be a particularly suitable candidate for CAD/CAM fabrication because, unlike most appliances, it needs no activatable element apart from its protrusion spring^23, 24^. Its design is instead intended to promote maxillary growth and redirect mandibular growth in order to reduce the need for subsequent orthognathic surgery^25^. It mainly affects maxillary growth in two ways. One, it retracts the soft tissue, disinhibiting sagittal and transversal maxillary growth, and two, it exerts a pull on the maxillary periosteum, leading to bone apposition. In tandem, the tight mandibular fit of the appliance inhibits mandibular translation and redirects mandibular growth in a dorsal direction^23, 26, 27^.

Because no studies describe a CAD/CAM-manufactured FR3 (CAD-FR3), no knowledge exists regarding its mechanical properties. However, this information is a basic requirement to determine the clinical applicability of such an appliance. This is particularly important because the conventional FR3 (Con-FR3) differs from most other orthodontic appliances in its extremely complex technical construction. The Con-FR3 is expensive to make and requires comprehensive knowledge from the dental technician. Accordingly, greater clinical use of the Con-FR3 is limited worldwide. Furthermore, the Con-FR3 is very fragile in its construction, which makes it susceptible to damage. Slight pressure on the palatal connector can lead to an inaccurate fit, which can in turn reduce or even fully negate the therapeutic effect.

The null hypothesis of the present study was, that Con-FR3s and CAD-FR3s in different dimensions will not differ with regard to their biomechanical properties.

## Materials and methods

### Impression taking and fabrication of models

Because digital impressions cannot yet be used for functional imaging of the vestibule, the CAD-FR3s were planned on plaster models (Hinrizit; Ernst Heinrichs, Goslar, Germany). The models were then digitised by use of an intraoral scanner (Trios 4; 3Shape, Copenhagen, Denmark) to undergo subsequent digital processing. The use of patient models and the study protocol were approved by the Ethics Committee of Heidelberg University (approval number: S-066/2021) in accordance with the ethical standards in the revised Declaration of Helsinki (1975) and its later revisions. Informed consent was obtained from all participants and their legal guardians.

### Digital design and manufacturing of the CAD-FR3

**Figure 1:**
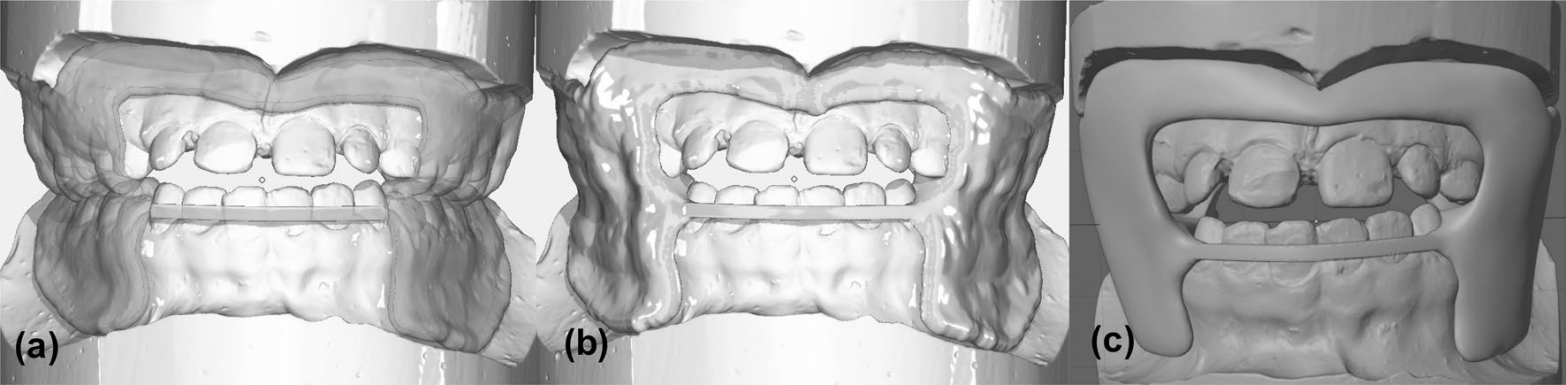
Stages of digital construction. (**a**) The maxillary and mandibular parts of each CAD/CAM function regulator 3 (CAD-FR3) were initially designed separately in the program OnyxCeph. (**b**) The two parts were then digitally fused together to form a complete appliance. (**c**) Finally, the exported surface tessellation files were post-processed in the software “Blender”.

The basic structure of all CAD-FR3s was initially constructed in two parts (maxilla and mandible) in the “Bite Splint” module of the software OnyxCeph (OnyxCeph; Image Instruments, Chemnitz, Germany; Fig. 1a). In accordance with the design described by Fränkel et al., the maxillary part was blocked out, and the mandibular part was constructed close to the bone. Moreover, the maxillary part was extended to end 2–3 mm apically of the vestibular fold. In contrast to the Con-FR3, the vestibular shield of the CAD-FR3 was designed as a single circular part. To increase both the stability and tension points on the maxillary periosteum, the shield was designed to extend 3 mm apically of the gingival margin.

After completion of the maxillary and mandibular parts, the palatal connector was designed with a distance to the palate of 2–4 mm. Then the labial archwire was constructed with a slight pre-activation. In the last construction step, all parts were fused together (Fig. 1b), and the overall design was exported as surface tessellation language (STL) files.

**Figure 2:**
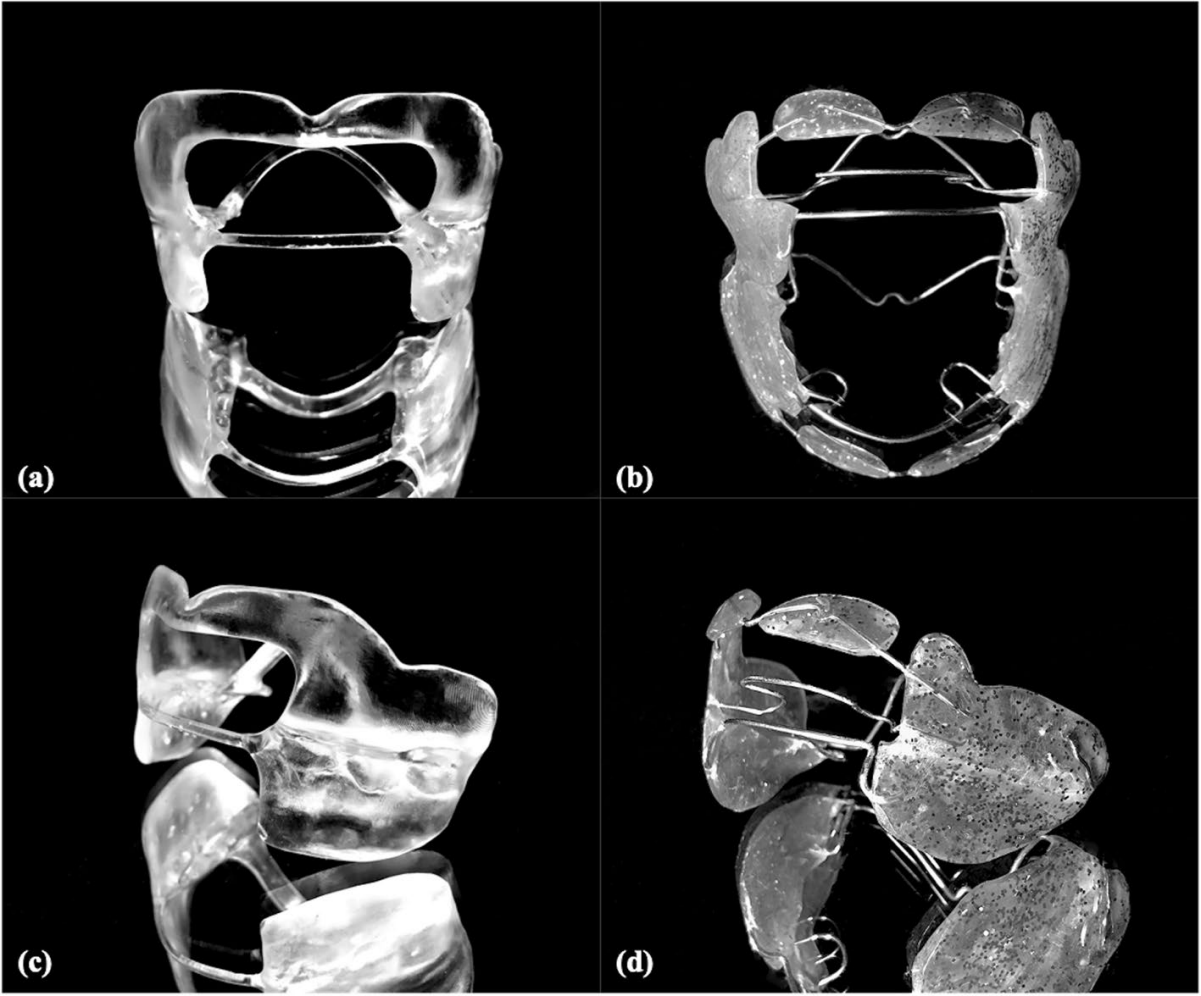
CAD/CAM function regulator 3 (CAD-FR3; **a**,**c**) and conventional FR3 (Con-FR3; **b**,**d**). Unlike the Con-FR3s, the CAD-FR3s had a vestibular shield of a one-piece design. In addition to increasing stability, this design was intended to increase the tension points on the periosteum and thus have an even greater effect on maxillary post-development. The CAD-FR3s were monolithic and therefore constructed with no metal elements.

**Figure 3:**
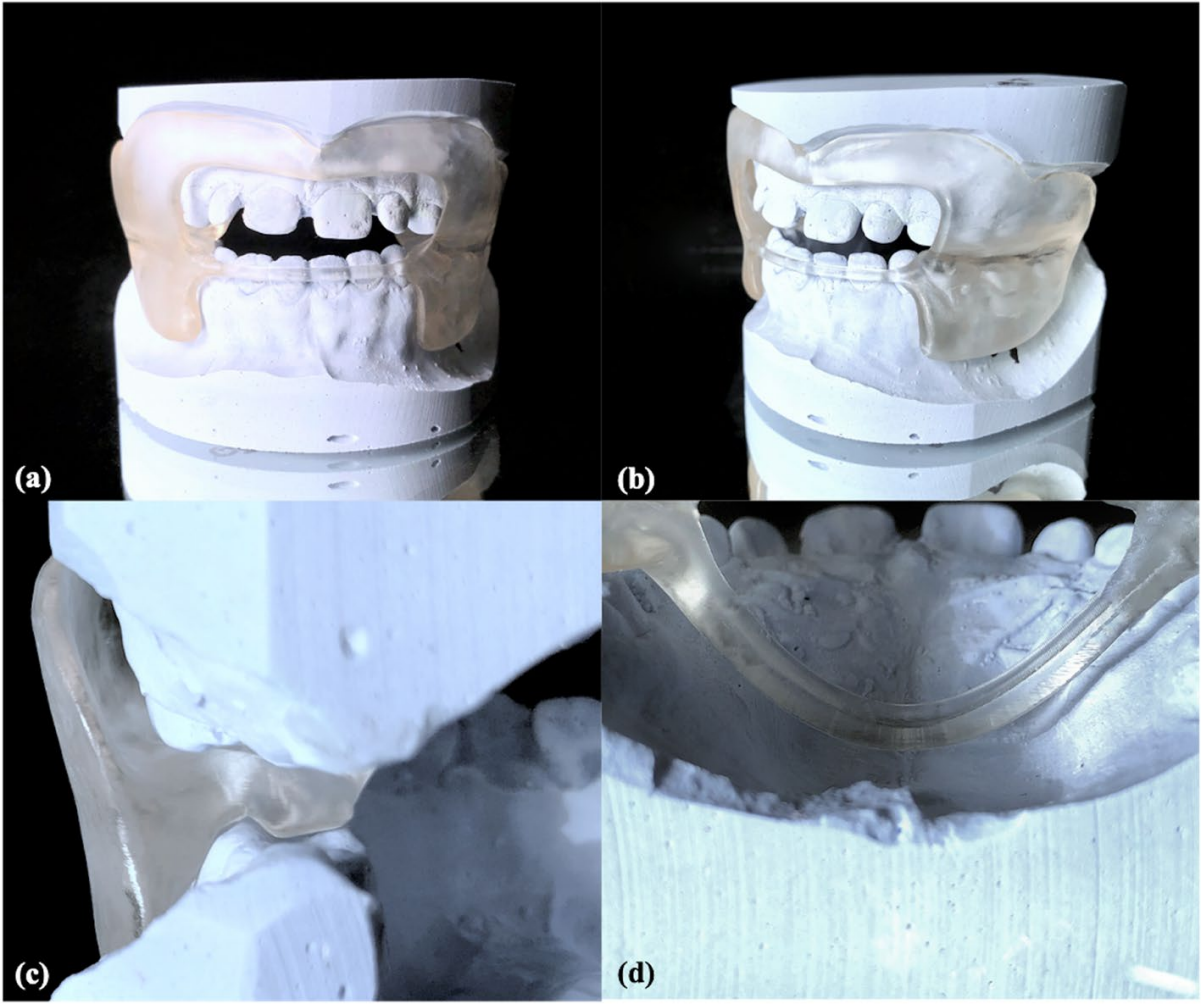
CAD/CAM functional regulator 3 (CAD-FR3) on plaster model. (**a**,**b**) To ensure maximum periosteal traction, CAD-FR3s were extended to the bottom of the maxillary vestibular fold, which had previously been deepened on the model by 2–3 mm along the alveolar ridge. In the maxilla, the vestibular parts were blocked out up to 3 mm, and the occlusal parts were flat in design to ensure unrestricted maxillary post-development. (**c**) In the mandible, both the vestibular and occlusal parts were designed to fit closely to the bone, to ensure maximum growth inhibition. (**d**) The palatal connectors were constructed 2–4 mm from the palate.

The CAD-FR3s were then digitally post-processed by use of Blender software (Blender; Blender Institute, Amsterdam, Netherlands; Fig. 1c). Essentially, this involved smoothing the edges and harmonising the surfaces of the STL files. In addition, because maximum maxillary offset in OnyxCeph was only 1 mm, the maxillary part was further blocked out up to 3 mm. To avoid introducing bias to the results, care was taken to ensure that the thickness of all parts remained unchanged and that the labial archwire and palatal connector were not modified by post-processing. To enable extrapolation of the study results to the clinical context, the shape and dimensions of the CAD-FR3s were designed to be clinically fully applicable and functional. Finally, all CAD-FR3s were printed with a 3D stereolithography printer (Form 3B; Formlabs, MA, USA; Figs. 2, 3). The resin used (Dental LT clear; Formlabs) was biocompatible and certified for the production of long-term intraoral appliances. After printing all CAD-FR3s were post-processed according to the manufacturer´s information, which means washing in Isopropyl alcohol (IPA) and then curing on 60 °C for 60 min.

### Pre-testing of CAD/CAM labial archwires of different dimensions

Before the transversal load capacity (F_max(FR3)_) of the FR3 appliances was tested, different CAD/CAM labial archwires (CAD-Ls) were pre-tested and compared with conventional labial archwires (Con-Ls). This pre-study was conducted to determine the optimum CAD-L design, which would then be implemented in all CAD-FR3s for subsequent transversal testing. The optimum archwire design should be as slim as possible but have sufficient load capacity (F_max(L)_). In addition, it should have stiffness values, that allow slight pre-activation of the wire within the design process.

**Figure 4:**
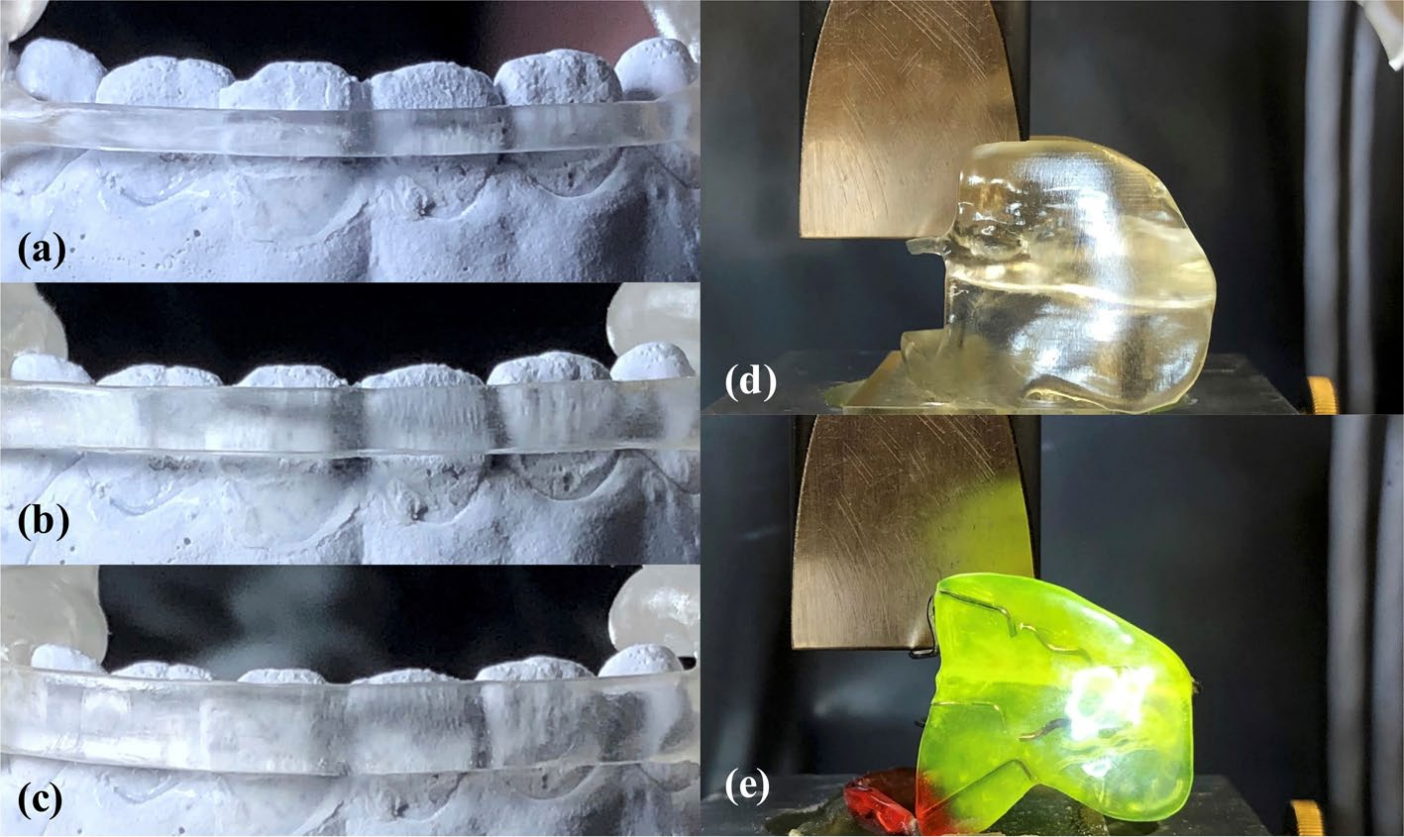
Testing of different labial CAD/CAM archwires. Before transversal load testing, three different labial CAD/CAM archwires (CAD-L) dimensions (**a**–**d**) were tested and compared to the conventional labial archwire (Con-L; **e**) in order to find a suitable CAD-L design that could be integrated in the final CAD/CAM functional regulator 3 designs for further transversal testing. The optimum CAD-L should be as slim as possible but have sufficient load capacity (Fmax(L)) and stiffness values comparable to those of the Con-L. All labial archwires were loaded caudally, to simulate resistance during insertion. Load was applied until mechanical failure was detected or until the deflection of the labial arch reached a predefined maximum value of 15 mm.

CAD-FR3s with three different CAD-L designs were constructed for each of ten patient cases (*n* female patients = 5). To minimise patient discomfort, all CAD-Ls were designed with a thickness of 1 mm, consistent with Con-L archwires. The height was increased from 2 to 4 mm in 1-mm increments (CAD-L_2–_L_4_; Fig. 4a–c). For pre-testing, all CAD-L designs were incorporated in the same CAD-FR3 design: CAD-FR3_5×2_ (palatal connector width and height: 5 and 2 mm, respectively).

All CAD-FR3s were fixed in a universal testing machine (Z005; Zwick Roell, Ulm, Germany) and loaded caudally to simulate resistance during insertion (Fig. 4d,e). An indenter with a tip radius of 1.6 mm was used to apply load vertically to the exact centre of the labial archwire. Load was applied until mechanical failure was detected or until the archwire reached a predefined maximum deflection of 15 mm. Anterior–posterior loading was not performed, as this was to be tested subsequently in the main transversal load test. Testing parameters were: pre-load: 1 N; cross-head speed up to pre-load: 5 mm/min; crosshead speed: 2 mm/min; fracture detection: 80% of the maximum test force. F_max(L)_ was defined as the maximum test force during the test and stiffness values were calculated by linear regression of test data in force displacement diagram between 0.5 and 0.6 mm. Both values were determined and compared among the different CAD-L designs and to the Con-L, respectively.

### Testing of palatal connectors of different dimensions during cyclic and ultimate transversal loading

**Figure 5:**
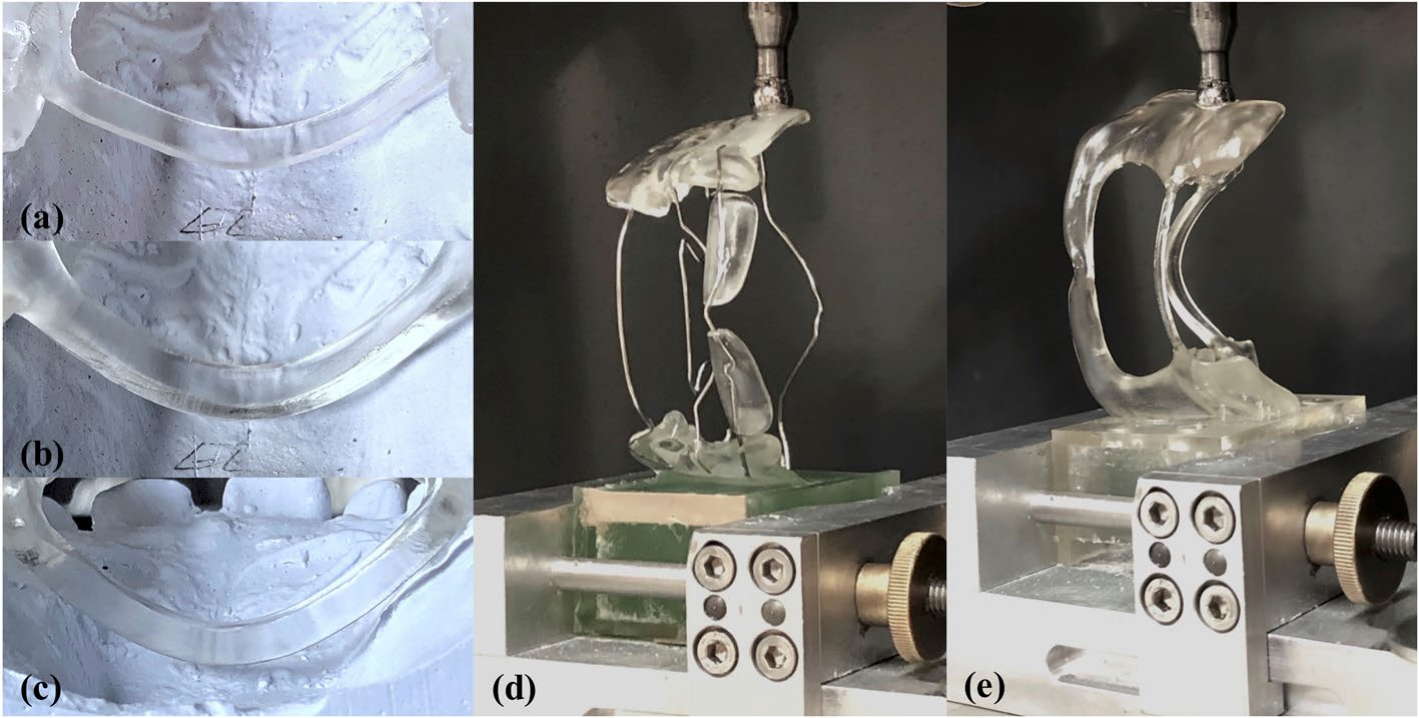
Transversal load testing of function regulator (FR3) designs. (**a**–**c**) CAD/CAM FR3s (CAD-FR3) were constructed with different palatal connector dimensions. Two connectors were constructed flat to the palate; one (**a**) had a width of 4 mm and a height of 1 mm (CAD-FR34 × 1) and the other one (**b**) had a width of 5 mm and a height of 2 mm (CAD-FR35 × 2). A third connector (**c**) was designed with a width and height of 3 mm (CAD-FR33 × 3). (**d**,**e**) All FR3 appliances underwent 1000 transversal load cycles at a load magnitude of 15 N before ultimate load testing was performed. Fmax(FR3) and stiffness values were compared among the different CAD-FR3 designs and conventional FR3s.

For the F_max(FR3)_ test, three CAD-FR3s with palatal connectors of different dimensions (Fig. 5a–c) were constructed for each of ten patient cases (*n* female patients = 4). The three sets of connector dimensions tested per patient were: width 4 mm, height 1 mm (CAD-FR3_4×1_); width 5 mm, height 2 mm (CAD-FR3_5×2_); width and height 3 mm (CAD-FR3_3×3_). The CAD-FR3s were digitally integrated into base sockets. Care was taken to ensure that only the buccal shield of all FR3s was integrated into the socket and that the palatal connector and archwire remained fully unobstructed. To maximise the reliability of transversal testing, an identical loading point was digitally constructed in the opposing, free buccal shield of all CAD-FR3s. Consistent with the Con-FR3s, in which loading points were milled as cavities during manual production, all CAD-FR3 loading points were constructed at the height of the palatal connector. The optimum CAD-FR3 design would be able to withstand daily insertion forces and retract the buccal muscles. For the F_max(FR3)_ test, the 2-mm-high labial archwire design (CAD-L_2;_ Fig. 4a) was integrated into all CAD-FR3s, because this design was the most slender and had the highest F_max(L)_ and lowest stiffness values of all pre-tested CAD-Ls.

To determine an appropriate force magnitude for cyclic loading, i.e. a magnitude corresponding to that exerted in clinical use, measurements were taken for several patients in clinical practice. Forces were measured during appliance insertion by means of a spring balance, and an upper threshold of 15 N was determined. Thus, all FR3 appliances underwent 1000 transversal load cycles at a magnitude of 15 N before ultimate load testing was performed (Fig. 5d,e). If premature failure occurred during cyclic load testing, the F_max(FR3)_ of the respective FR3 was recorded as 15 N. F_max(FR3)_ and stiffness values were determined and compared among the different CAD-FR3 designs and Con-FR3. Testing parameters were: pre-load: 1 N; pre-force speed up to pre-load: 5 mm/min; crosshead speed (cyclic load): 5 N/s; crosshead speed (ultimate load): 10 mm/min; fracture detection: 30% decline in F_max(FR3)_. F_max(FR3)_ was defined as the maximum test force during ultimate load test and stiffness values were calculated by linear regression of test data in force displacement diagram from last 10 transversal load cycles. Both values were determined and compared among the different CAD-FR3 designs and to the Con-FR3, respectively.

Moreover, to investigate the effect of CAD-FR3 size, which depended on the jaw size of the respective patient, the anterior–posterior distance (APd) was measured digitally from the loading point to the most anterior point of the respective FR3. These values were then correlated with the results of F_max(FR3)_ and stiffness testing.

### Statistical analysis

Data were analysed by use of the software program SPSS statistics 25 (IBM; Endicott, NY, USA). Data were compared among the different CAD-FR3 designs (each n = 10) and the Con-FR3 group (n = 10), respectively between the different CAD-L designs (each n = 10) and the Con-L group (n = 10). Sample size for each test was planned in consultation with the Institute of Biometry and Informatics of the XXX University. Due to the sample size non-parametric tests were chosen. Since the same 10 patient situations were used for the main test and other same 10 patient situations were used for the pre-tests, test data between test groups were statistically dependent. Consequently, Friedman and two-tailed Wilcoxon tests were used to identify differences between the test groups for the respective test. Statistical significance was set at p < 0.05.

### Ethics approval and consent to participate

This research project was approved by the ethics committee of the University of Heidelberg (approval number: S-066/2021). Informed consent was obtained from all participants and their legal guardians.

## Results

### Results of pre-tests on labial archwires: F_max(L)_ and stiffness

**Figure 6:**
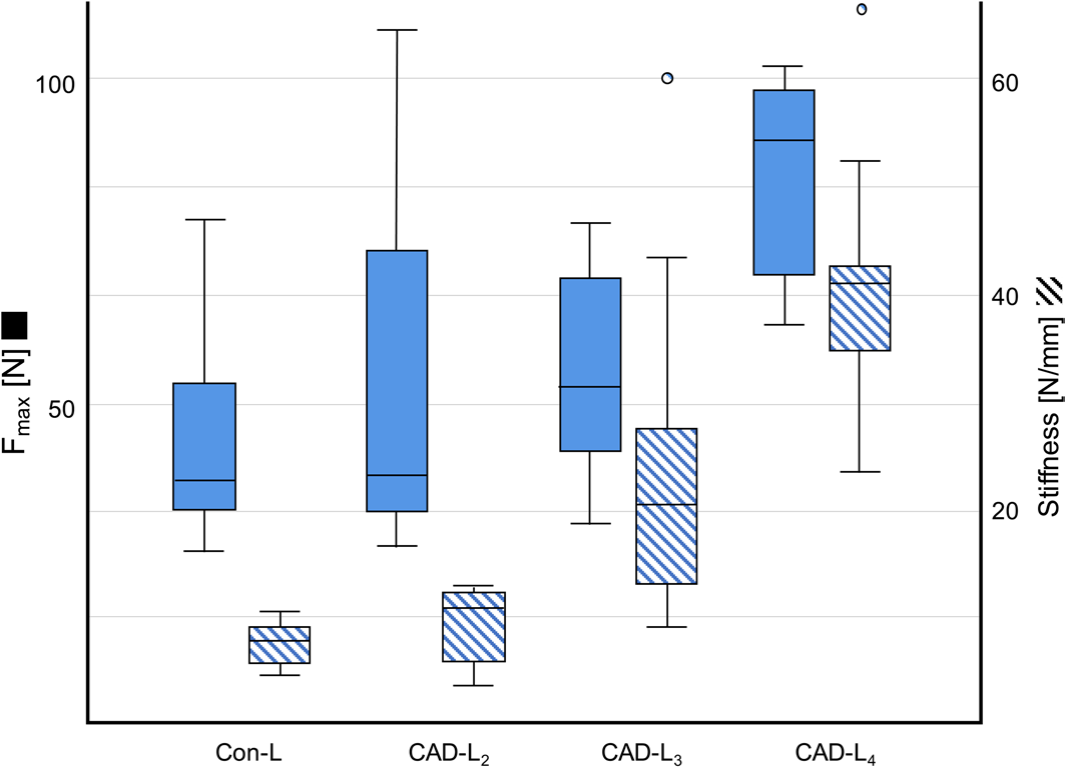
Results of pre-tests on conventional labial archwires (Con-L) and CAD/CAM labial archwires (CAD-L). All CAD-L groups had a higher mean load capacity (F_max(L)_) and higher mean stiffness than the Con-Ls. CAD-L_2_ was therefore determined to be the optimum archwire, because it had the slimmest design and provided sufficient F_max(L)_. Moreover, because of its similar stiffness values in comparison to the Con-L group, it should allow slight pre-activation of the labial arch. Consequently, CAD-L_2_ was implemented in all CAD-FR3 designs for further transversal testing.

The Con-L group had a mean F_max(L)_ of 46.0 ± 17.1 N (Fig. 6). All three CAD-L groups had a higher mean F_max(L)_ than the Con-L group, which was statistically significant for CAD-L_4_ (p ≤ 0.005). Mean F_max(L)_ was 53.6 ± 30.3 N for the CAD-L_2_ group, 54.6 ± 14.5 N for the CAD-L_3_ group and 86.1 ± 14.7 N for the CAD-L_4_ group.

A mean stiffness of 7.6 ± 1.9 N/mm was recorded for the Con-L group. Mean stiffness in the CAD-L_2_ group was similar (9.2 ± 3.6 N/mm). The CAD-L_3_ and CAD-L_4_ groups had significantly (*p* ≤ 0.005) higher mean stiffness values of 24.2 ± 16.3 N/mm and 41.4 ± 11.8 N/mm, respectively. When forces over F_max(L)_ were applied, this resulted in the breakage of all CAD-Ls and the plastic deformation of Con-L. Because CAD-L_2_ had sufficient F_max(L)_ and similar stiffness values in comparison to the Con-L group, this design was incorporated in all CAD-FR3s for further transversal load testing.

### Results of main tests: transversal load capacity and stiffness

**Figure 7:**
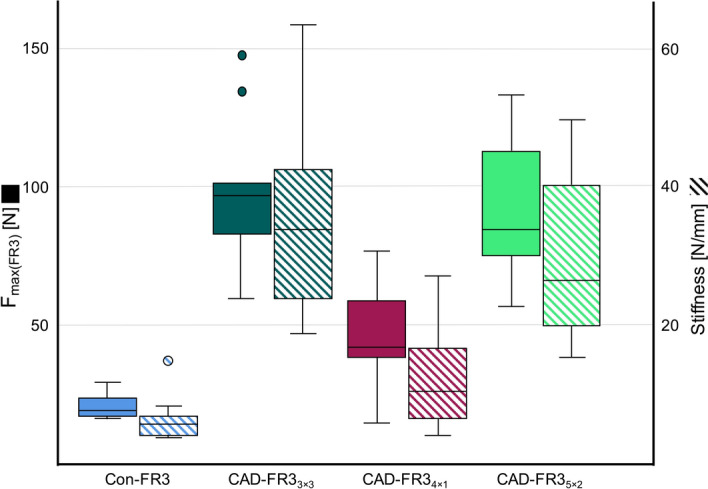
Results of transversal testing. All CAD/CAM function regulator 3 (CAD-FR3) groups had a higher mean load capacity (F_max(FR3)_) than the conventional FR3 group (Con-FR3). The mean F_max(FR3)_ of CAD-FR3_3×3_ and CAD-FR3_5×2_ were nearly five times higher than that of Con-FR3. CAD-FR3_4×1_ had the lowest mean F_max(FR3)_ of all CAD-FR3 designs, and two individual cases even had the lowest F_max(FR3)_ of all FR3s tested. All CAD-FR3 groups had higher mean stiffness than the Con-FR3 group. Load application above F_max(FR3)_ caused most CAD-FR3s to break, whereas in all the Con-FR3s this resulted in plastic deformation.

The Con-FR3 group had a mean F_max(FR3)_ of 20.7 ± 7.2 N (Fig. 7). In comparison, the mean F_max(FR3)_ values of all CAD-FR3 groups were significantly (*p* ≤ 0.007) higher. The highest mean F_max(FR3)_, which was 98.2 ± 26.2 N, was recorded for the CAD-FR3_3×3_ group. The mean F_max(FR3)_ for the CAD-FR3_5×2_ group, which was 90.3 ± 24.7 N, was only slightly lower. In contrast, the CAD-FR3_4×1_ group had a mean F_max(FR3)_ of 45.8 ± 17.9 N, which was significantly (*p* ≤ 0.007) lower than that of the other two CAD-FR3 designs, although still two times higher than that of the Con-FR3 group. However, looking at the individual cases, two CAD-FR3_4×1_ had the lowest F_max(FR3)_ of all FR3s tested, reaching values of 15.0 N and 28.8 N. One of these CAD-FR3_4×1_ broke at the stage of cyclic testing.

Similar to the trend observed for F_max(FR3)_, all CAD-FR3 groups had significantly (*p* ≤ 0.005) higher mean stiffness than the Con-FR3 group (Fig. 7). For Con-FR3s, mean stiffness was 6.3 ± 3.4 N/mm. Stiffness values for the CAD-FR3 groups were 37.1 ± 15.5 N/mm for CAD-FR3_3×3_, 30.0 ± 12.3 N/mm for CAD-FR3_5×2_ and 12.5 ± 7.3 N/mm for CAD-FR3_4×1_. Load application above F_max(FR3)_ caused most CAD-FR3s to break, whereas in all the Con-FR3s this resulted in plastic deformation.

**Figure 8:**
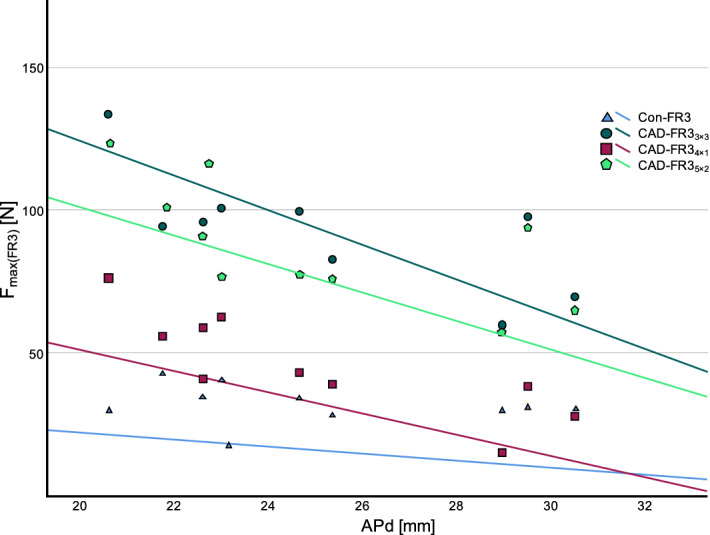
Load capacity (Fmax(FR3)) of function regulator 3 (FR3) designs in relation to anterior–posterior distance (APd). APd was measured from the loading point to the most anterior point of the respective FR3. All CAD/CAM FR3s (CAD-FR3s) showed a greater APd-dependent decrease of Fmax(FR3) than conventional FR3s (Con-FR3s). However, despite their greater APd-dependent decrease, all CAD-FR35 × 2 and CAD-FR33 × 3 still had higher Fmax(FR3) than the other groups.

**Figure 9:**
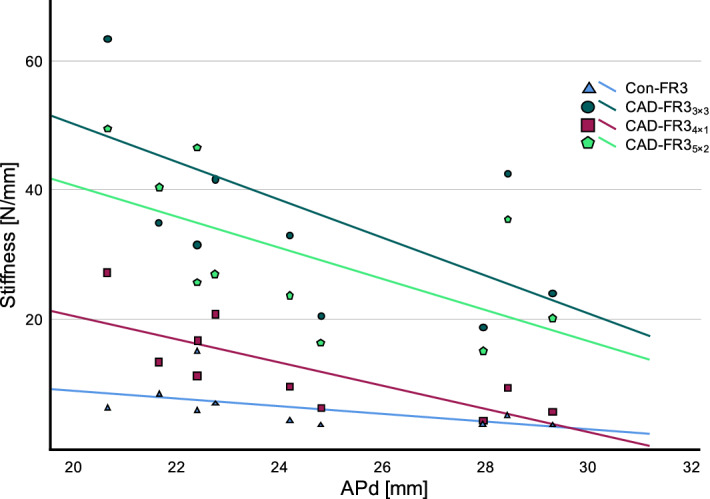
Stiffness of function regulator 3 (FR3) designs in relation to anterior–posterior distance (APd). As observed for load capacity (Fmax(FR3)), all CAD/CAM FR3s (CAD-FR3s) showed a greater APd-dependent decrease of stiffness than conventional FR3s (Con-FR3s).

All CAD-FR3 groups showed a greater APd-dependent decrease of F_max(FR3)_ and stiffness than Con-FR3s (Figs. 8 and 9). However, despite this greater APd-dependent decrease, all individual CAD-FR3_5×2_ and CAD-FR3_3×3_ still had higher F_max(FR3)_ than the other groups.

## Discussion

The null hypothesis had to be rejected, because the results of the present study showed that CAD-FR3s differed from Con-FR3s regarding F_max(FR3)_ and stiffness. The CAD-FR3_3×3_ had the highest mean F_max(FR3)_ and stiffness values, both of which were almost five times higher than those for the Con-FR3. However, the mean F_max(FR3)_ and stiffness of the CAD-FR3_5×2_ were only slightly lower. As a result, and because its palatal connector has a flatter design that potentially provides greater wearing comfort, the CAD-FR3_5×2_ might be particularly suitable for clinical use. In contrast, the mean F_max(FR3)_ and stiffness values for the CAD-FR3_4×1_ were significantly (*p* ≤ 0.007) lower, and two individual cases had the lowest F_max(FR3)_ of all FR3s tested. One CAD-FR3_4×1_ even broke during cyclic stress testing. This design therefore appears to be clinically unusable.

Moreover, the results of the pre-tests suggest that it might be possible to integrate slender CAD-Ls into the final CAD-FR3 design. Low stiffness values combined with a sufficient F_max(L)_ were found for the CAD-L_2_ design. Thus, this design might enable slight pre-activation of the labial arch to be included in the digital construction process, as is recommended for the Con-L^24^.

This is the first study to describe a CAD-FR3. So far, CAD/CAM technology has mainly been used for aligners^6, 7^ and fixed orthodontic appliances^10, 11^; it has very rarely been described in relation to functional orthopaedic appliances. This might primarily be because it is difficult to fuse the metal elements and plastic base of functional appliances by means of CAD/CAM technology. Nevertheless, Al Mortadi et al. demonstrated the CAD/CAM production of an Andresen activator^21^. However, their study included no biomechanical stress tests. It therefore remains unclear whether the activator would be sufficiently stable for clinical use. The concept proposed by Al Mortadi et al. seems interesting, but its realisation remains complicated. For example, individual guiding jigs, which are required to construct the metal elements of the activator must be constructed and printed in advance. Moreover, in order to insert the metal elements, which must be bent and adapted to the guiding jigs, the printing of the activator base must be paused. Therefore, the method described by Al Mortadi et al. does not permit fully automated manufacture of the activator.

Another CAD/CAM concept, for the production of an active plate, was presented in a proof-of-concept study by van der Meer et al.^22^. In this study, metal parts were not produced manually, but by a bending robot. This fully digital planning and manufacturing approach could be very promising. Nevertheless, the difficulties posed by fusing metal and plastic elements again means that it remains challenging to implement this concept in everyday clinical practice. Although the wire elements are bent completely digitally, they must be bonded manually to the base plate by a technician after printing.

Both studies explore interesting concepts regarding how functional orthodontic appliances might be manufactured in the future by using CAD/CAM techniques. However, both concepts use a manufacturing process that seems rather time-consuming and complicated and therefore needs further simplification before it can be integrated into clinical routine. Nevertheless, both concepts could have potential for the CAD/CAM realisation of orthodontic appliances that necessarily require activatable metal elements.

All CAD-FR3s in this study were constructed without any metal elements. In contrast to most functional orthodontic appliances, however, a metal-free design might be feasible for the FR3. This is because all its metal components, except for the protrusion spring, only provide stability and must not be activated^23^. Instead, the focus is on an optimum fit, in order to achieve the best possible therapeutic effect in terms of inhibited mandibular translation or dorsally directed growth redirection and uninhibited maxillary post-development^28^. It is therefore important that the maxillary and mandibular fit of the appliance remain unchanged throughout treatment. Regarding its maxillary effect in particular, the FR3 is only efficient if its palatal connector can withstand buccal forces to create enough functional space and ensure sufficient periosteal traction. In this regard, the CAD-FR3 might be superior to the Con-FR3: 28 of 30 CAD-FR3s had higher transversal stiffness values than the Con-FR3, which might mean that the CAD-FR3 can achieve more constant periosteal traction and therefore a greater maxillary effect. Conversely, this higher transversal stiffness led to a higher probability of breakage in the CAD-FR3s, whereas forces exceeding F_max(FR3)_ in the Con-FR3s led only to plastic deformation. Nonetheless, the F_max(FR3)_ that caused the CAD-FR3s to break were three times higher than those that caused the Con-FR3s to deform. Intraoral breakage of the CAD-FR3 is therefore rather unlikely. Extraoral breakage, in contrast, might uncover when the CAD-FR3 is non-functional, whereas the plastic deformation of a Con-FR3 could initially go unnoticed. This seems particularly important, because both the maxillary and the mandibular part must fit precisely to achieve the optimum therapeutic effect. Conversely, higher transversal stiffness might complicate the insertion of the FR3. However, the aim of this in-vitro study was solely to determine whether further clinical testing of the CAD-FR3 would be justified. Consequently, the effect of increasing transversal stiffness on clinical applicability and therapeutic effect can only be answered by further clinical trials.

The CAD-FR3 does not contain a protrusion spring. In most cases, however, the protrusion spring is only required in the initial stage of FR3 therapy to transfer the frontal crossbite. Normal frontal bite relation should be achieved as quickly as possible, so that the blocking of the FR3 in the molar region can be reduced to 1.5 mm^23^. This should reduce lip closure obstruction and ensure the wearing comfort of the appliance. Therefore, the short-term use of other appliances such as active plates before CAD-FR3 therapy might be conceivable, because they apply force to the incisors in a faster and more targeted way.

The Con-FR3 is a very complicated appliance to produce. Consequently, its manufacture requires specially trained technicians. These factors have prevented the Con-FR3 from being used more widely. This study sought to determine whether CAD/CAM-manufactured FR3s might be a viable alternative to Con-FR3s. If so, this could provide dentists throughout the world (including those without access to a trained technician) with another means of producing FR3s, and thus facilitate the wider use of this appliance. Many dentists could benefit from a simple chairside production of the FR3. To make this a reality, however, the production process requires further simplification. But for the CAD-FR3 in particular this seems feasible. Only the design process of the concept presented requires simplification; the manufacturing process can already be performed completely automatically. In the future, it might be possible to automatically digitally project the FR3 onto previously digitised models, thus enabling fast and easy chairside production of the CAD-FR3. The possibility of digitally archiving and processing both the model and construction data might also offer further advantages. Model archiving would require no physical storage space. Moreover, the digital models could be used to analyse therapeutic effects. For example, digitally matching patient models of different therapy stages or projecting models into lateral cephalograms or three-dimensional facial photographs, as already used for diagnostics and surgical planning^29, 30^, might enable more precise planning of the FR3. Saving the construction data could provide further advantages. Adjustments could be quickly and easily implemented in the saved construction data in order to print a new design, an option that would not be possible for a Con-FR3. Furthermore, should the appliance be lost or damaged, the dentist could re-print the archived design and insert it after about 1.5 h’ printing time. As a result, the patient would be without their appliance for a shorter amount of time. This appears to be very important, because complication rates for inflexible removable appliances are up to 25%^31, 32^. Faster reintegration might also have a positive effect on therapy, because the time taken for a conventional repair negatively affects therapy time and therapeutic effect by 16% and 9%, respectively^33, 34^.

Several limitations should be considered when interpreting the results of this study. First, biomechanical cyclic load force and direction can vary from one patient to another. However, load force for cyclic stress testing was first determined by means of a spring balance when inserting Con-FR3s on several patients in everyday clinical practice, and then set particularly high at 15 N. Second, because an in-vitro setting can only partly represent the clinical situation, it is impossible to draw specific conclusions concerning the in-vivo implications of our results.

Nonetheless, this study shows for the first time that FR3s can be constructed in CAD/CAM technology and that their technical properties can be superior to those of Con-FR3s if certain design parameters are followed. These results justify further clinical investigations. Further studies are now required to determine whether CAD-FR3s are also comparable to Con-FR3s regarding patient acceptance and therapeutic effect and can therefore be used in everyday clinical practice.

## Conclusions

Within the limitations of a laboratory study, the results demonstrate that CAD-FR3s have superior mechanical properties in comparison to Con-FR3s if certain design parameters are considered. Because the Con-FR3 requires a particularly sophisticated production technique, the widespread use of this appliance remains limited. Therefore, both dentists and patients might benefit from having access to a CAD/CAM alternative.

## Data Availability

Data available on request. The data underlying this article will be shared on reasonable request to the corresponding author.
